# Aerosol-Assisted Metal Poisoning of Cu/SSZ-13 during NH**_3_**‑SCR Operation

**DOI:** 10.1021/acs.est.6c07432

**Published:** 2026-08-31

**Authors:** Tao Zhang, Jianning Zhang, Feng Yu, Qi Dong, Jian Liu, Junhua Li, Evgenii V. Kondratenko

**Affiliations:** † School of Chemistry and Life Resources, 12471 Renmin University of China, Beijing 100872, P. R. China; ‡ Department of Advanced Methods for Applied Catalysis, 28392 Leibniz-Institut für Katalyse e.V. (LIKAT), Rostock 18059, Germany; § College of Science, China University of Petroleum, Beijing 102249, P. R. China; ∥ College of Chemistry and Chemical Engineering, 47846 Taiyuan University of Technology, Taiyuan 030024, P. R. China; ⊥ School of Petrochemical Engineering, Liaoning Petrochemical University, Fushun 113001, P. R. China; # State Key Joint Laboratory of Environment Simulation and Pollution Control, School of Environment, 12442 Tsinghua University, Beijing 100084, P. R. China

**Keywords:** NH_3_−SCR, Cu/SSZ-13, metal poisoning, aerosol-assisted poisoning, reaction-coupled deactivation

## Abstract

Cu/SSZ-13 is a benchmark catalyst for the selective catalytic reduction (SCR) of NO_*x*_ with NH_3_ in diesel exhaust aftertreatment, but its activity can be severely impaired by alkali and alkaline-earth metal poisoning. Conventional impregnation of catalysts with such poisons involves introducing metal species prior to catalytic testing. This means that any poisoning that develops concurrently with catalyst operation is not captured. Here, we develop an aerosol-assisted, reaction-coupled poisoning platform that continuously delivers metal-containing aqueous aerosols to SCR catalysts under hydrated NH_3_–SCR conditions. At nearly identical K loadings, aerosol-assisted poisoning caused substantially greater deactivation of Cu/SSZ-13 than conventional impregnation of the catalyst, with a more pronounced activity loss after poisoning at 400 °C than at 200 °C. The enhanced deactivation was accompanied by a more spatially extensive K distribution, stronger perturbations of the zeolite framework, preferential alteration of 8-membered-ring-associated [Cu­(OH)]^+^–Z species, spectroscopic signatures consistent with CuO_*x*_ aggregation, and partial neutralization of Brønsted acid sites. Similar pathway- and temperature-dependent deactivation trends were observed for Na-, Mg-, and Ca-poisoned Cu/SSZ-13 and for K-poisoned V_2_O_5_–WO_3_/TiO_2_. These results establish aerosol-assisted poisoning as a controlled, accelerated approach for evaluating metal-induced deactivation of SCR catalysts under hydrated reaction conditions.

## Graphical abstract

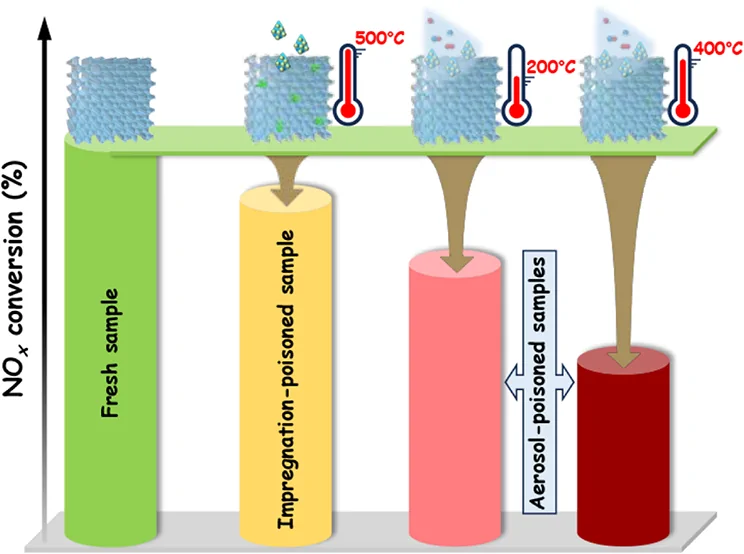

## 1 Introduction

Cu/SSZ-13 zeolite catalysts are widely recognized as state-of-the-art materials for NO_*x*_ abatement via the selective catalytic reduction (SCR) of NO_*x*_ with NH_3_ in diesel exhaust aftertreatment.^1–5^ Despite their high intrinsic activity and hydrothermal stability, these catalysts undergo progressive deactivation under practical operating conditions.^6–8^ Among the various causes of deactivation, poisoning by alkali (Na, K) and alkaline-earth (Mg, Ca) metals represents a particularly persistent challenge.^9,10^ These metals can originate from several sources associated with diesel operation, including fuel-derived impurities, especially in biodiesel applications, lubricant oil additives, commercial urea solutions, and atmospheric aerosol particulates, the latter being especially relevant in coastal environments.^11–14^

In practical diesel exhaust aftertreatment systems, catalyst deactivation usually develops gradually over extended time scales, often over years, which makes direct laboratory-scale mechanistic studies difficult. Accelerated deactivation protocols using artificially increased concentrations of alkali and alkaline-earth metal poisons are therefore commonly used as a pragmatic compromise. Most studies on metal poisoning of Cu/SSZ-13 rely on ex situ impregnation, in which metal species are introduced from liquid-phase precursors, followed by drying and calcination.^15–19^ The resulting deactivation behavior is then evaluated by comparing fresh and prepoisoned catalysts, and poisoning mechanisms are inferred from these post-treatment differences.

Such static prepoisoning treatments, however, separate metal introduction from catalyst operation. During service, metal-bearing aerosol or gas-phase species can reach the catalyst while it is exposed to varying temperature, water content, and reaction atmosphere.^7^ Under these conditions, metal deposition, transport within the hydrated catalyst, and interaction with catalytically relevant sites may evolve concurrently with the SCR reaction. Conventional impregnation therefore may not fully capture how the poisoning pathway influences framework integrity, Brønsted acidity, Cu speciation, and the resulting loss of SCR activity.

To address this gap, we developed an aerosol-assisted poisoning protocol that continuously introduces alkali and alkaline-earth metal species into the reactant stream during ongoing NH_3_–SCR. The protocol enables accelerated, reaction-coupled poisoning under controlled laboratory conditions and is intended to capture poisoning processes that occur during catalyst operation rather than to reproduce the full duration and complexity of real-world aging. Using K-poisoned Cu/SSZ-13 as the primary model system, we compare aerosol-assisted poisoning with conventional impregnation at nearly identical K loadings and relate the resulting changes in SCR performance to changes in framework structure, Cu speciation, and Brønsted acid functionality. The approach is further extended to Na-, Mg-, and Ca-poisoned Cu/SSZ-13 and to K-poisoned commercial V_2_O_5_–WO_3_/TiO_2_ to assess whether the effects of the metal-introduction pathway and poisoning temperature persist across different metal poisons and SCR catalyst architectures.

## 2 Experimental Section

### 2.1 Catalyst Preparation

Fresh Cu/SSZ-13 catalyst (Si/Al = 13.5, 2.67 wt % Cu; denoted CZ_F) was supplied by China Catalyst Holding Co., Ltd.

Fresh V_2_O_5_–WO_3_/TiO_2_ catalyst (1 wt % V, 4 wt % W; denoted V–W/Ti_F) was prepared by incipient wetness impregnation. In a typical synthesis, H_2_C_2_O_4_ was dissolved in deionized water, followed by the addition of NH_4_VO_3_ and (NH_4_)_6_H_2_W_12_O_40_ under stirring until complete dissolution. TiO_2_ (P25) was then added to the solution, and the resulting suspension was stirred at 80 °C for 2 h. The mixture was dried at 110 °C for 12 h and subsequently calcined in air at 500 °C for 4 h.

Alkali- and alkaline-earth-metal-poisoned catalysts were prepared using an in-house aerosol-assisted, reaction-coupled poisoning setup, as schematically illustrated in Figure 1b. Aerosols were generated from aqueous solutions of the corresponding metal nitrates using a Yuwell 402AI ultrasonic nebulizer (Jiangsu Yuyue Medical Equipment & Supply Co., Ltd., China). According to the manufacturer’s technical manual, the nebulizer produces droplets with a nominal volume-equivalent diameter of 3.9 μm and a stated tolerance of ± 25%. This specification was determined by the manufacturer using 0.9 wt % saline at 20 ± 5 °C and 50 ± 10% relative humidity.

**Figure 1:**
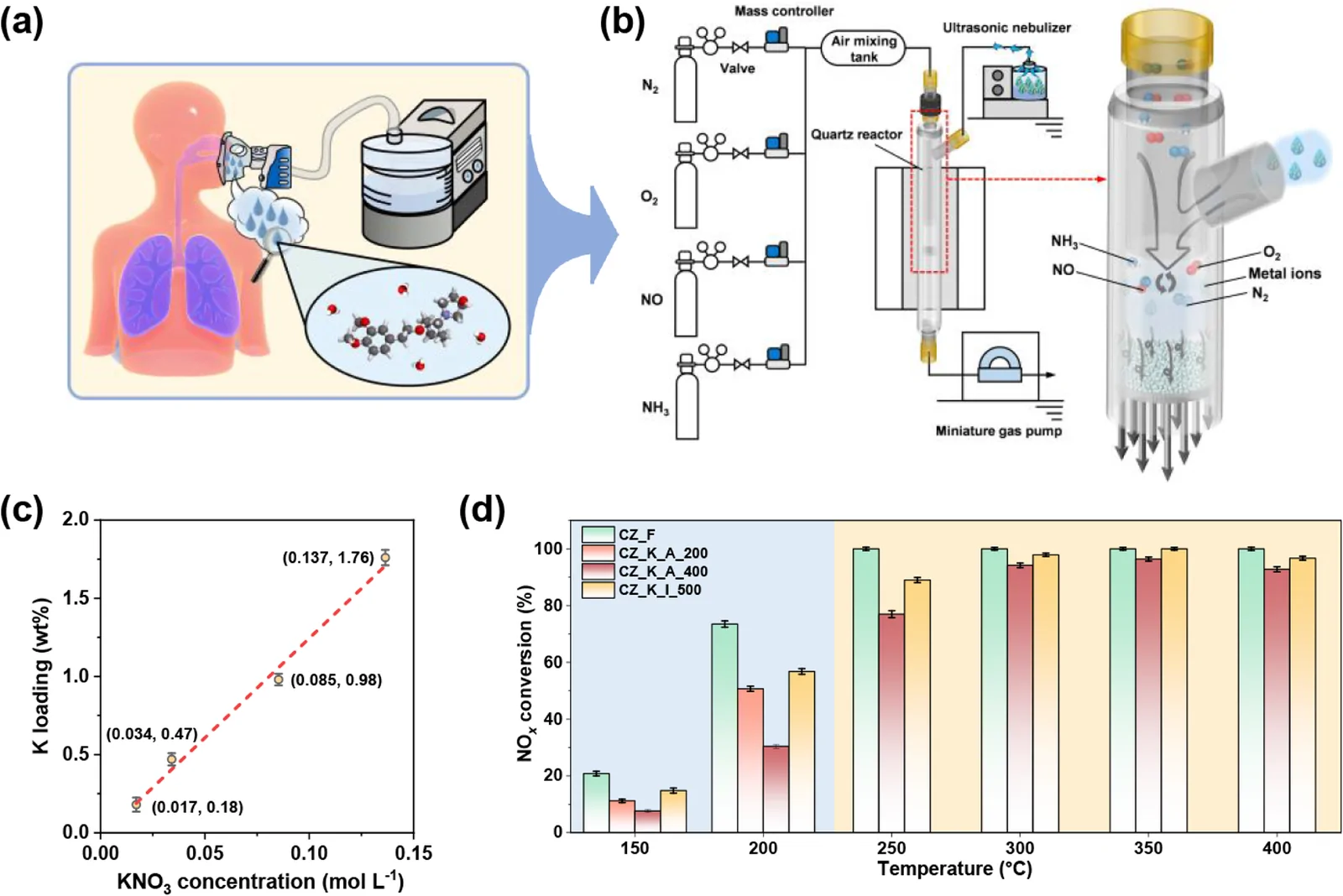
Aerosol-assisted, reaction-coupled alkali-metal poisoning of Cu/SSZ-13 and the resulting NH_3_–SCR performance. (a) Schematic illustration of nebulization-based drug delivery. (b) Aerosol-assisted poisoning system comprising an ultrasonic nebulizer coupled to a fixed-bed reactor operated under NH_3_–SCR conditions. (c) K loading as a function of the KNO_3_ concentration in the nebulizer solution. (d) NO*_x_* conversion as a function of reaction temperature for the fresh, aerosol-poisoned, and impregnated K-poisoned samples. In panels (c) and (d), the data points represent the mean of three independent measurements, and the error bars indicate one standard deviation.

The poisoning atmosphere contained 500 ppm of NH_3_, 500 ppm of NO, and 5 vol % O_2_ in N_2_. The operating power of the nebulizer was adjusted to control the amount of aqueous aerosol introduced into the reactor such that the water delivered with the aerosol corresponded to 5 vol % H_2_O in the total feed, matching the H_2_O content used in the standard NH_3_–SCR activity tests. Thus, the aerosol simultaneously introduced the metal species and established the hydrated SCR atmosphere. For each treatment, 0.5 g of catalyst and a total gas flow rate of 500 mL min^–1^ were used. Poisoning was conducted at either 200 or 400 °C for 12 h. The two temperatures were selected to represent distinct SCR operating regimes: 200 °C is relevant to low-load operation and periods before the exhaust system reaches its higher-temperature regime, whereas 400 °C represents operation under elevated engine load. Except for the poisoning temperature and the concentration of the metal nitrate solution, all other aerosol-introduction parameters, including the total gas flow rate, catalyst mass, treatment duration, nebulizer operating power, and resulting H_2_O concentration, were kept constant. The target metal loading was adjusted by varying the concentration of the corresponding metal nitrate in the nebulizer solution, while the actual metal content was subsequently quantified by inductively coupled plasma optical emission spectrometry (ICP-OES).

The resulting aerosol-poisoned samples are denoted CZ_M_A_T and V–W/Ti_M_A_T, where M identifies the introduced metal, A denotes aerosol-assisted poisoning, and T indicates the poisoning temperature. To evaluate possible contributions from nitrate-derived species, control treatments were performed using HNO_3_ solutions with the same total nitrate concentration as the corresponding metal nitrate solutions under otherwise identical conditions. The resulting control samples are denoted CZ_NO_3_^–^_A_T, where T indicates the treatment temperature.

For the K mass-balance analysis, the total amount of K introduced into the system was determined from the decrease in the K inventory of the nebulizer reservoir during the poisoning treatment. The amount of K retained by the catalyst was quantified by ICP-OES after complete acid digestion. Potassium deposited on the quartz wool, aerosol-transfer line, reactor inlet, reactor wall, downstream tubing, and outlet collection system was quantitatively recovered by rinsing, and the resulting solutions were analyzed by ICP-OES.

For comparison, metal-poisoned catalysts were also prepared by conventional incipient wetness impregnation using the corresponding metal nitrate solutions as precursors. After impregnation, the samples were dried at 110 °C for 12 h and calcined in air at 200, 400, or 500 °C for 4 h. These samples are denoted CZ_M_I_T and V–W/Ti_M_I_T, where I denotes conventional impregnation and T indicates the calcination temperature.

### 2.2 Catalytic Activity Tests

The NH_3_–SCR performance of the fresh and poisoned catalysts (40–60 mesh) was evaluated in a fixed-bed quartz-tube reactor. The feed consisted of 500 ppm of NH_3_, 500 ppm of NO, 5 vol % O_2_, and 5 vol % H_2_O, with N_2_ as the balance gas. For the Cu/SSZ-13 catalysts, 0.02 g of catalyst was tested at a total flow rate of 200 mL min^–1^, corresponding to a gas hourly space velocity (GHSV) of 400,000 h^–1^. For the V_2_O_5_–WO_3_/TiO_2_ catalysts, 0.2 g of catalyst was tested at the same total flow rate, corresponding to a GHSV of 60,000 h^–1^. The outlet gas composition was continuously monitored using an online Fourier transform infrared (FTIR) gas analyzer (Thermo Scientific Antaris IGS). NO_*x*_ conversion and N_2_ selectivity were calculated using the following equations:

1 $${\mathrm{NO}}_{x}\mathrm{conversion}\;\left(\%\right)=\frac{{[{\mathrm{NO}}_{x}]}_{\mathrm{inlet}}-{[{\mathrm{NO}}_{x}]}_{\mathrm{outlet}}}{{[{\mathrm{NO}}_{x}]}_{\mathrm{inlet}}}\times 100$$

2 $$N_{2}\;\mathrm{selectivity}\;\left(\%\right)=\left(1-\frac{2{[N_{2}O]}_{\mathrm{outlet}}}{{[{\mathrm{NO}}_{x}]}_{\mathrm{inlet}}+{[{\mathrm{NH}}_{3}]}_{\mathrm{inlet}}-{[{\mathrm{NO}}_{x}]}_{\mathrm{outlet}}-{[{\mathrm{NH}}_{3}]}_{\mathrm{outlet}}}\right)\times 100$$

### 2.3 Catalyst Characterization

The catalysts were characterized in terms of elemental composition, morphology, crystalline structure, textural properties, local framework structure, Cu speciation, redox behavior, and surface-adsorbed species by inductively coupled plasma optical emission spectroscopy (ICP-OES), transmission electron microscopy (TEM) with energy-dispersive X-ray spectroscopy (EDS) elemental mapping, X-ray photoelectron spectroscopy (XPS), powder X-ray diffraction (PXRD), N_2_ physisorption, solid-state magic-angle-spinning nuclear magnetic resonance (MAS NMR), electron paramagnetic resonance (EPR), H_2_ temperature-programmed reduction (H_2_-TPR), and in situ diffuse reflectance Fourier transform infrared spectroscopy (DRIFTS). Details of the characterization methods are provided in the Supporting Information.

## 3 Results and Discussion

### 3.1 Development and Quantitative Validation of the Aerosol-Assisted Poisoning Platform

Inspired by nebulization-based drug delivery (Figure 1a), we developed an aerosol-assisted, reaction-coupled poisoning platform that continuously introduces metal-containing aerosols into an SCR catalyst bed under reaction conditions (Figure 1b). In contrast to conventional impregnation, in which metal species are introduced before catalytic testing, this platform couples metal introduction and deposition with ongoing NH_3_–SCR operation, allowing poisoning to proceed within a hydrated catalyst bed under working conditions.

Potassium was selected as the primary model poison because it is commonly used in conventional impregnation-based poisoning studies of Cu/SSZ-13 ^9,17^ with a loading of about 1 wt %, providing an established basis for comparing the two poisoning protocols. Across three independent experiments, nebulization of a 0.085 mol L^–1^ KNO_3_ solution reproducibly produced an average catalyst K content of approximately 0.98 wt % (Figure 1c). The solution contained 8.59 g L^–1^ KNO_3_, corresponding to approximately 0.86 wt %. Based on the solution consumption during the 12 h treatment and the total wet-gas volume, the nominal inlet mass concentrations were calculated to be 315 mg m^–3^ for KNO_3_ and 122 mg m^–3^ for elemental K.

The aerosol was generated at a laboratory temperature of approximately 25 °C and a relative humidity of approximately 40%. These conditions were close to those used by the manufacturer to characterize the nebulizer with 0.9 wt % saline at 20 ± 5 °C and 50 ± 10% relative humidity. The generated droplets were therefore expected to remain within the same micrometer-size regime as the manufacturer-reported nominal volume-equivalent diameter of 3.9 μm with a stated tolerance of ± 25%.

To evaluate the controllability of the aerosol-assisted poisoning approach, the KNO_3_ concentration in the nebulizer solution was systematically varied. The catalyst K content increased monotonically with increasing KNO_3_ concentration (Figure 1c), showing that the final K loading could be systematically adjusted through the precursor concentration.

The quantitative performance was further evaluated by a system-wide K mass-balance analysis. Across three independent experiments, approximately 11% of the introduced K was retained by the catalyst, while the overall K mass-balance closure was approximately 96%. The retained K consistently produced the target catalyst loading of approximately 1 wt %, and the high mass-balance closure confirmed that nearly all introduced K was recovered from the catalyst and the aerosol-delivery and reactor components. Together, these results establish the reproducibility, controllability, and quantitative consistency of the aerosol-assisted poisoning platform.

### 3.2 Pathway- and Temperature-Dependent SCR Deactivation

Following quantitative validation of the platform, we examined how the metal-introduction pathway and treatment temperature affected NH_3_–SCR performance. All K-poisoned samples showed lower NO_*x*_ conversion than the fresh catalyst over the investigated temperature range (Figure 1d), whereas N_2_ selectivity was affected to a much smaller extent (Figure S1). Notably, at nearly identical K loadings (approximately 0.98 wt % on average for the aerosol-poisoned samples versus 1.00 wt % for the conventionally impregnated sample), both aerosol-poisoned catalysts exhibited lower NO_*x*_ conversion than the conventionally impregnated catalyst. In addition, aerosol-assisted poisoning at 400 °C caused more severe activity loss than poisoning at 200 °C, showing that the poisoning temperature affected the extent of K-induced deactivation during NH_3_–SCR operation. A similar, though less pronounced, trend was observed for the conventionally impregnated catalysts (Figure S2).

Control experiments were then conducted to assess possible contributions from the aerosol treatment itself and from nitrate-derived species. After 12 h of exposure to a pure-water aerosol at either 200 or 400 °C, both fresh Cu/SSZ-13 and the conventionally impregnated K-poisoned catalyst showed no appreciable loss in NO_*x*_ conversion (Figure S3). Likewise, the HNO_3_ control treatments did not produce deactivation comparable to that observed after aerosol-assisted K poisoning (Figure S4). These results show that the pronounced activity loss of the aerosol-poisoned samples cannot be explained by nitrate-derived species or by the combined effects of aerosol exposure, hydrated NH_3_–SCR operation, and thermal treatment in the absence of aerosol-delivered K.

To further assess whether external mass-transfer limitations or reactor-scale bed effects contributed to the apparent activity differences, additional NH_3_–SCR measurements were performed at 130 °C under low-conversion conditions over a GHSV range of 200,000–800,000 h^–1^. NO_*x*_ conversion decreased with increasing GHSV because of the shorter gas residence time, while the activity order CZ_F > CZ_K_I_500 > CZ_K_A_200 > CZ_K_A_400 was preserved over the entire range (Figure S5a). Moreover, the apparent first-order rate coefficient, *k*_app_, calculated using a plug-flow approximation, remained nearly constant for each catalyst, deviating by less than 3% from the corresponding value at 400,000 h^–1^ (Figure S5b). The absence of a systematic dependence on GHSV indicates that the measured activity differences are not dominated by external mass-transfer limitations or gross reactor-scale bed effects.

### 3.3 Spatial Distribution of Potassium and Associated Framework Perturbation

To probe the structural basis of the distinct deactivation behavior described above, we first examined the spatial distribution of potassium and the framework structure of the poisoned catalysts. EDS elemental mapping showed a relatively uniform K distribution across individual crystallites of the aerosol-poisoned samples, whereas K-rich regions were concentrated near the crystallite peripheries of the conventionally impregnated sample (Figure 2a). XPS provided complementary information on the near-surface composition. At closely matched bulk K contents, the near-surface K atomic concentration followed the order CZ_K_I_500 (0.60 at. %) > CZ_K_A_200 (0.46 at. %) > CZ_K_A_400 (0.38 at. %) (Table S1). The higher value for CZ_K_I_500 is consistent with the peripheral K enrichment observed by EDS. By comparison, the lower near-surface K concentration of the aerosol-poisoned samples, together with their more uniform EDS distribution, indicates less preferential accumulation of K near the crystallite peripheries and a broader spatial distribution across the crystallites. The further decrease from 0.46 at. % for CZ_K_A_200 to 0.38 at. % for CZ_K_A_400 suggests that near-surface K enrichment becomes less pronounced as the poisoning temperature increases.

**Figure 2:**
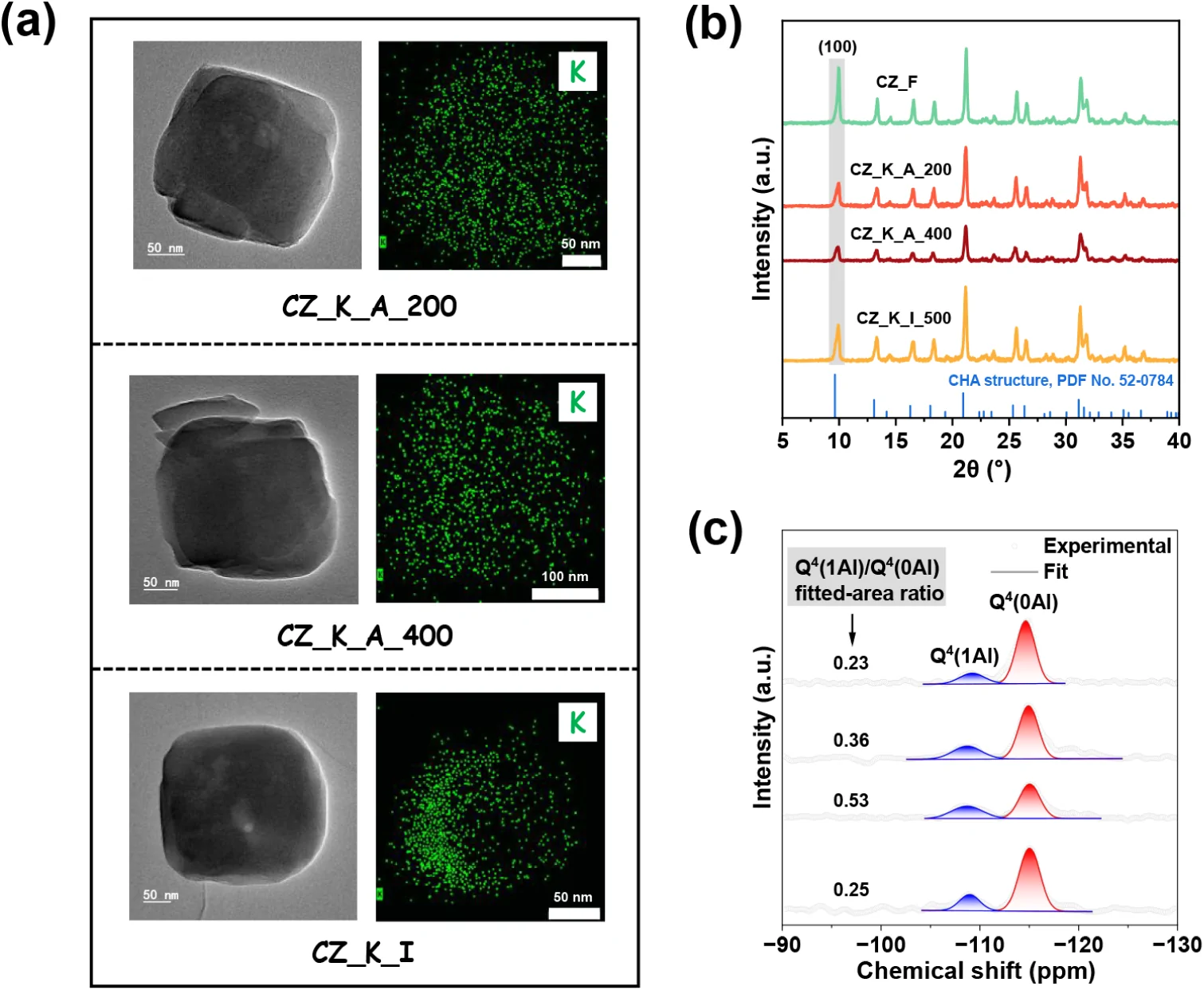
Spatial distribution of K and framework perturbation in Cu/SSZ-13 after different potassium poisoning protocols. (a) TEM images and corresponding EDS elemental maps of K for the aerosol-poisoned and impregnated K-poisoned samples. (b) PXRD patterns and (c) ^29^Si MAS NMR spectra of the fresh, aerosol-poisoned, and impregnated K-poisoned samples.

All samples retained the characteristic reflections of the chabazite (CHA) topology (PDF No. 52–0784), indicating that the overall SSZ-13 framework remained largely preserved after potassium poisoning (Figure 2b). No secondary crystalline phases were detected. However, the aerosol-poisoned samples showed pronounced attenuation of the CHA reflections, with a greater decrease for CZ_K_A_400, suggesting a partial loss of long-range crystallographic order. By contrast, the conventionally impregnated sample showed marked attenuation mainly of the CHA (100) reflection, which has been associated with structural changes involving the 8-membered-ring (8MR) windows.^20,21^ N_2_ physisorption measurements further showed a progressive decrease in specific surface area after potassium poisoning (Figure S6 and Table S1), consistent with the accompanying textural and crystallographic changes.

To examine the local framework environment, ^27^Al and ^29^Si MAS NMR measurements were performed. The ^27^Al MAS NMR spectra of the fresh and K-poisoned samples were dominated by a resonance at 57.7–59.0 ppm, characteristic of tetrahedrally coordinated framework Al (Al_f_), whereas no pronounced signal attributable to octahedrally coordinated extra-framework Al near 0 ppm was detected (Figure S7).^22^ Compared with CZ_F, the aerosol-poisoned samples showed a small but systematic shift of the framework-Al resonance maximum toward higher chemical shift, with a larger shift for CZ_K_A_400 than for CZ_K_A_200. No discernible shift was observed for CZ_K_I_500. Because the ^27^Al resonance position is sensitive to the local charge-compensating and hydration environment, these changes indicate a more pronounced modification of the local environment around framework Al during aerosol-assisted K poisoning.^23^

The ^29^Si MAS NMR spectra were dominated by resonances centered near −115.0 and −109.0 ppm, assigned to Q^4^(0Al) and Q^4^(1Al) environments, respectively (Figure 2c).^24^ Because the two resonances partially overlap, they were deconvoluted using two Gaussian functions under identical fitting conditions for all samples. The resulting semiquantitative Q^4^(1Al)/Q^4^(0Al) fitted-area ratio increased from 0.23 for CZ_F to 0.36 and 0.53 for CZ_K_A_200 and CZ_K_A_400, respectively, whereas CZ_K_I_500 retained a value of 0.25. The substantially larger changes observed for the aerosol-poisoned samples indicate a stronger alteration of the relative contributions of the two local Si environments than that produced by conventional impregnation.^25^ No pronounced resonances attributable to partially condensed Q^3^ or Q^2^ Si species were observed, indicating that these local changes were not accompanied by extensive Si-framework depolymerization.

The combined EDS/XPS, PXRD, and MAS NMR results show that aerosol-assisted poisoning is associated with a more spatially extensive K distribution and greater perturbations of both long-range crystallographic order and local Al and Si environments than conventional impregnation, with the most pronounced changes observed for CZ_K_A_400.

### 3.4 Changes in Framework-Associated Cu Species and Acid Functionality

The broader spatial distribution of K and the associated perturbation of the zeolite framework are expected to affect the Cu species responsible for NH_3_–SCR activity. Cu/SSZ-13 generally contains two principal framework-associated Cu species: isolated Cu^2+^ ions located at 6-membered rings (6MRs), denoted Cu^2+^–Z_2_, and [Cu­(OH)]^+^–Z species located near the 8-membered-ring (8MR) windows of CHA cages, where Z denotes a negatively charged framework Al site (Figure 3a). We therefore examined the effects of K poisoning on Cu speciation and acid functionality.

**Figure 3:**
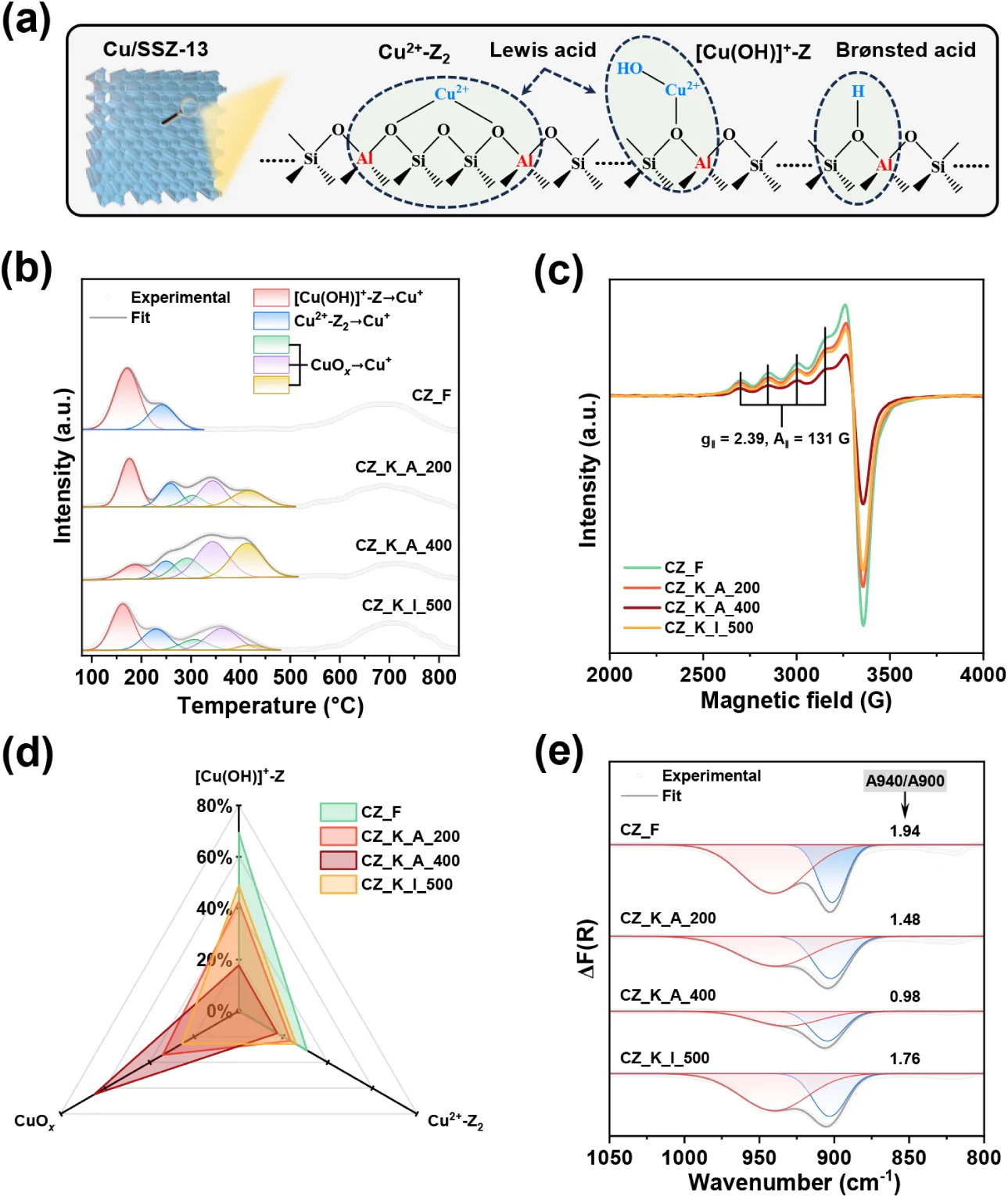
Evolution of Cu species in Cu/SSZ-13 under different potassium poisoning protocols. (a) Schematic representation of the Cu^2+^–Z_2_, [Cu­(OH)]^+^–Z, and Brønsted acid sites in the CHA framework. (b) H_2_-TPR profiles with corresponding peak deconvolution for the fresh, aerosol-poisoned, and impregnated K-poisoned samples. (c) EPR spectra of Cu^2+^ species in the hydrated fresh, aerosol-poisoned, and impregnated K-poisoned samples. (d) Comparison of relative changes in Cu speciation derived from quantitative analyses based on Table S1. (e) NH_3_-DRIFTS spectra in the 1050–800 cm^–1^ range along with the corresponding band deconvolution for the fresh, aerosol-poisoned, and impregnated K-poisoned samples.

For the fresh catalyst, H_2_-TPR revealed two H_2_-consumption maxima below 500 °C (Figure 3b), which are assigned to the reduction of [Cu­(OH)]^+^–Z and Cu^2+^–Z_2_ species to Cu^+^, respectively.^26,27^ The H_2_ consumption above 500 °C is attributed to the subsequent reduction of Cu^+^ to Cu^0^. This assignment is supported by the ratio of H_2_ consumption below and above 500 °C (Figure S8). Upon K poisoning, the reduction features associated with framework-related [Cu­(OH)]^+^–Z and Cu^2+^–Z_2_ species were attenuated, while three additional reduction events emerged in the 300–500 °C range. These new features are consistent with the reduction of aggregated CuO_*x*_ species to Cu^0^ (Figure S9). Their multipeak character suggests the presence of CuO_*x*_ aggregates with heterogeneous local coordination environments,^28^ likely formed through displacement of framework-associated Cu species by K^+^, followed by Cu migration and aggregation (Figure S8). The resulting CuO_*x*_ species may also reduce micropore accessibility, consistent with the decrease in specific surface area (Table S1). Their absence from the PXRD patterns (Figure 2b) does not preclude their formation, because the CuO_*x*_ species may be X-ray amorphous or, if crystalline, below the detection limit of PXRD.

Consistent with the H_2_-TPR results, EPR spectra collected under hydrated conditions show pronounced attenuation of the signal intensity assigned to paramagnetic Cu^2+^ ions (g_||_ = 2.39, A_||_ = 131 G) after K poisoning (Figure 3c).^29^ NH_3_-DRIFTS measurements likewise showed substantial attenuation of the band at 1613 cm^–1^, which is mainly associated with Lewis acid sites of isolated Cu^2+^ species (Figure S10a).^30^ These observations support a decrease in framework-associated Cu species. The band at 1485 cm^–1^ also decreased after K poisoning, consistent with partial neutralization of Brønsted acid sites (BASs) in Cu/SSZ-13 (Figure S10a).^30^ BASs can contribute to NH_3_–SCR by storing adsorbed NH_3_ and thereby sustaining its availability near Cu redox sites.^22^ They have also been implicated in acid-assisted transformation of reaction intermediates generated at Cu sites (Figure S10b).^31,32^ Partial neutralization of BASs may therefore further contribute to SCR deactivation by weakening NH_3_ storage and the cooperative interaction between Cu and acid sites.

Quantitative analysis of the H_2_-TPR profiles revealed that the Cu-related H_2_ consumption area decreased more for the aerosol-poisoned catalysts than for the conventionally impregnated catalyst. The largest decrease was observed after poisoning at 400 °C (Figure 3d). The contribution assigned to reduction of [Cu­(OH)]^+^–Z species also decreased more strongly than that assigned to Cu^2+^–Z_2_ species. A consistent trend was observed by NH_3_-DRIFTS (Figure 3e). The negative IR bands at approximately 940 and 900 cm^–1^, assigned to [Cu­(OH)]^+^–Z and Cu^2+^–Z_2_ species, respectively, showed greater attenuation for the aerosol-poisoned catalysts than for the conventionally impregnated catalyst, with the most pronounced changes observed for CZ_K_A_400. Because these two bands partially overlap, their relative contributions were evaluated by Gaussian deconvolution. The resulting semiquantitative fitted-area ratio, A940/A900, decreased from 1.94 for the fresh catalyst to 1.48 for CZ_K_A_200 and 0.98 for CZ_K_A_400, whereas CZ_K_I_500 retained a higher value of 1.76. The progressive decrease in A940/A900 indicates that the relative spectroscopic contribution associated with [Cu­(OH)]^+^–Z decreased more strongly than that associated with Cu^2+^–Z_2_ during aerosol-assisted K poisoning.

The combined H_2_-TPR and NH_3_-DRIFTS results identify a pronounced pathway-dependent vulnerability of 8MR-associated [Cu­(OH)]^+^–Z species, with the strongest alteration observed after aerosol-assisted poisoning at 400 °C. Their preferential loss or transformation, together with partial neutralization of Brønsted acid sites, provides a structural basis for the substantially greater low-temperature SCR deactivation caused by aerosol-assisted K poisoning than by conventional impregnation.^33,34^

### 3.5 Generality Across Metal Poisons and SCR Catalysts

To examine the broader applicability of the aerosol-assisted poisoning platform, the protocol was extended from K to Na, Mg, and Ca using Cu/SSZ-13 as the catalyst. It was further applied to K poisoning of a V_2_O_5_–WO_3_/TiO_2_ catalyst, representing an oxide-based NH_3_–SCR system. At comparable metal loadings, aerosol-assisted poisoning of Cu/SSZ-13 with Na, Mg, or Ca caused greater deactivation than conventional impregnation, consistent with the trend observed for K (Figure 4, S10, and S11). Poisoning at 400 °C also generally resulted in more severe deactivation than poisoning at 200 °C, although the magnitude of the activity loss varied with the identity of the metal species. K-poisoned V_2_O_5_–WO_3_/TiO_2_ showed the same overall dependence on the metal-introduction pathway and poisoning temperature. The recurrence of these trends across monovalent and divalent metal poisons and across zeolite- and oxide-based SCR catalysts shows that the enhanced deactivation associated with aerosol-assisted delivery is not unique to the K/Cu-SSZ-13 model system. Rather, these results highlight the importance of the metal-introduction pathway and poisoning temperature in determining catalyst deactivation under hydrated NH_3_–SCR conditions. The aerosol-assisted platform therefore provides a controlled protocol for the accelerated evaluation of metal poisoning across different SCR catalyst systems.

**Figure 4:**
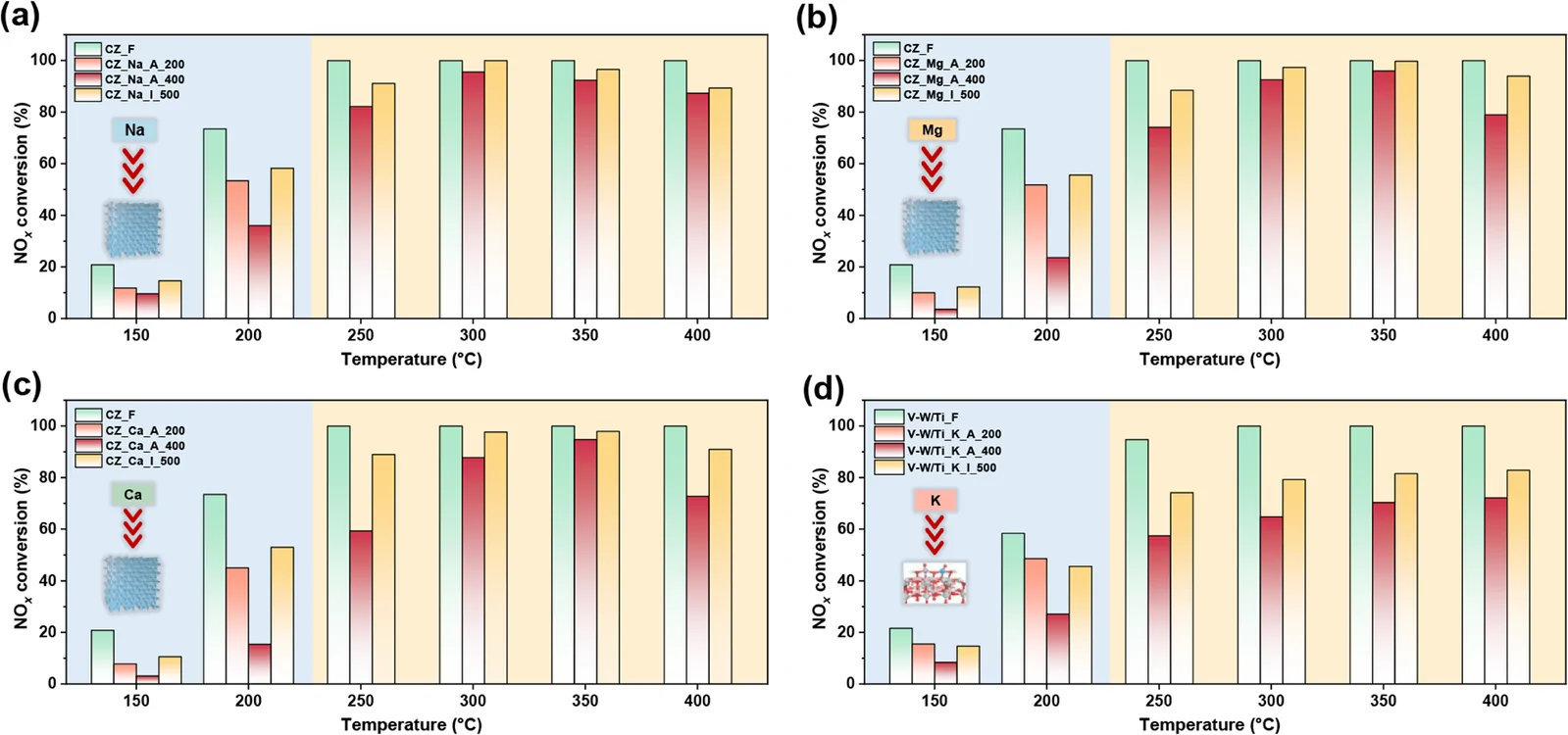
Generality of aerosol-assisted poisoning across different metal ions and catalyst systems. NO*_x_* conversion versus reaction temperature over fresh, aerosol-poisoned, and impregnated poisoned catalysts: (a) Na-, (b) Mg-, and (c) Ca-poisoned Cu/SSZ-13; (d) K-poisoned V_2_O_5_–WO_3_/TiO_2_.

## 4 Environmental Implications

Durable NH_3_–SCR catalysts are essential for sustained NO_*x*_ emission control. However, conventional ex situ poisoning protocols may not fully capture how alkali and alkaline-earth metal species are introduced, distributed, and interact with catalysts during hydrated SCR operation. Here, we establish an aerosol-assisted, reaction-coupled poisoning platform that continuously delivers metal species to SCR catalysts during ongoing NH_3_–SCR while maintaining the required H_2_O concentration in the feed.

For Cu/SSZ-13, aerosol-assisted K poisoning caused substantially greater deactivation than conventional impregnation at nearly identical K loadings, particularly when poisoning was conducted at 400 °C. The greater activity loss was accompanied by a more spatially extensive K distribution, stronger perturbations of the zeolite framework, greater alteration of 8MR-associated [Cu­(OH)]^+^–Z species, spectroscopic evidence consistent with CuO_*x*_ aggregation, and partial neutralization of Brønsted acid sites. These results show that poisoning severity is governed not only by the total Kloading but also by the K-introduction pathway and poisoning temperature.

The preferential alteration of low-temperature Cu species highlights the importance of preserving accessible [Cu­(OH)]^+^–Z sites and the local ion-exchange environment when designing more durable Cu/SSZ-13 catalysts. The extension of the protocol to Na-, Mg-, and Ca-poisoned Cu/SSZ-13 and to K-poisoned V_2_O_5_–WO_3_/TiO_2_ further demonstrates its applicability across different metal poisons and SCR catalyst architectures. The aerosol-assisted platform therefore provides a controlled and accelerated approach for evaluating metal-induced catalyst deactivation under hydrated NH_3_–SCR conditions.

## Supplementary Material



## Notes

Catalyst characterization methods, supplementary catalytic performance data, TEM/EDS mapping, N_2_ physisorption, H_2_-TPR deconvolution and reference profiles, NH_3_-DRIFTS spectra, ICP-determined metal contents, and additional textural and Cu speciation data (PDF)
