# Supplementary material for: Aerosol-Assisted Metal Poisoning of Cu/SSZ-13 during NH 3 ‑SCR Operation
Source: Environ Sci Technol. 2026 Aug 31;60(40):28577–85. doi: 10.1021/acs.est.6c07432 (PMC13674061; doi:10.1021/acs.est.6c07432)
Supplement: Supplementary file 1 [file es6c07432_si_001.pdf]

Supporting Information

Aerosol-Assisted Metal Poisoning of Cu/SSZ-13 during NH_3-SCR Operation

Tao Zhang,[1,2,#,] Jianning Zhang,[1,3,#] Feng Yu,[4,#] Qi Dong,[4] Jian Liu,[3,5,*] Junhua Li,[6] and Evgenii V. Kondratenko[2,*]*

[1] School of Chemistry and Life Resources, Renmin University of China, Beijing 100872, P. R. China

[2] Department of Advanced Methods for Applied Catalysis, Leibniz-Institut für Katalyse e.V. (LIKAT), Rostock 18059, Germany

[3] College of Science, China University of Petroleum, Beijing 102249, P. R. China

[4] College of Chemistry and Chemical Engineering, Taiyuan University of Technology, Taiyuan 030024, P. R. China

[5] School of Petrochemical Engineering, Liaoning Petrochemical University, Fushun 113001, P. R. China

[6] State Key Joint Laboratory of Environment Simulation and Pollution Control, School of Environment, Tsinghua University, Beijing 100084, P. R. China

[#] T.Z., J.Z., and F.Y. contributed equally to this work.

* Corresponding Authors, E-mail: zhangt@ruc.edu.cn; evgenii.kondratenko@catalysis.de; liujian@cup.edu.cn

18 pages, 12 figures, and 1 table.

Table of Contents

1. Catalyst Characterization Methods S3

2. Supplementary Figures.. S5

3. Supplementary Tables S17

References S18

1. Catalyst Characterization Methods

Inductively coupled plasma optical emission spectroscopy (ICP-OES) measurements were performed on an Agilent 730 ICP-OES instrument.

Transmission electron microscopy (TEM) and energy-dispersive X-ray spectroscopy (EDS) elemental mapping were conducted on an FEI Tecnai G2 F20 analytical electron microscope operated at an accelerating voltage of 200 kV.

X-ray photoelectron spectroscopy (XPS) measurements were performed on a Thermo Fisher Scientific ESCALAB 220iXL spectrometer using monochromatic Al K_α radiation (E = 1486.6 eV). Electron binding energies were measured with charge compensation provided by a flood electron source and calibrated against the C 1s peak of adventitious carbon at 284.8 eV.

Powder X-ray diffraction (XRD) patterns were recorded on a Shimadzu XRD-6000 diffractometer using Cu Kα radiation (λ = 1.54178 Å). Data were collected over a 2θ range of 5-90° at a scan rate of 10° min^{-1}.

N_2 physisorption measurements were carried out on a Micromeritics TriStar II 3020 analyzer at −196 °C using N_2 as the adsorbate. The specific surface area was calculated using the Brunauer–Emmett–Teller (BET) method.

Solid-state ^{27}Al and ^{29}Si magic-angle-spinning nuclear magnetic resonance (MAS NMR) spectra were acquired on a 14.1 T JEOL JNM-ECZ600R spectrometer equipped with a 3.2 mm HXY probe. The ^{27}Al MAS NMR spectra were recorded at 156.23 MHz using single-pulse direct excitation with a 30° pulse, a recycle delay of 0.1 s, 2000 scans, and a MAS rate of 15 kHz. The ^{29}Si MAS NMR spectra were recorded at 119.32 MHz using single-pulse

direct excitation with a recycle delay of 20 s and a MAS rate of 10 kHz. The same acquisition parameters were applied to all samples within each nucleus.

Electron paramagnetic resonance (EPR) spectra were collected on a Bruker A300-10/12 spectrometer. Signals of Cu^{2+} species were recorded at 155 K with a magnetic field sweep from 2000 to 4000 G.

H_2 temperature-programmed reduction (H_2-TPR) experiments were performed on a Micromeritics AutoChem 2920 instrument equipped with a thermal conductivity detector (TCD). Typically, 100 mg of sample was pretreated in He (30 mL min^{-1}) at 300 °C for 1 h, cooled to 50 °C, and then exposed to 10% H_2/He (50 mL min^{-1}). The temperature was ramped at 10 °C min^{-1} to 850 °C. Reduction peaks were quantified by Gaussian peak deconvolution.

In situ diffuse reflectance Fourier transform infrared (DRIFTS) spectra were recorded on a Bruker INVENIO S FTIR spectrometer equipped with an MCT/A detector and a diffuse-reflection optical system. Samples were pretreated at 500 °C for 30 min in 5% O_2/N_2 prior to background collection. Spectra were collected at a resolution of 2 cm^{-1} with 100 scans per spectrum.

2. Supplementary Figures

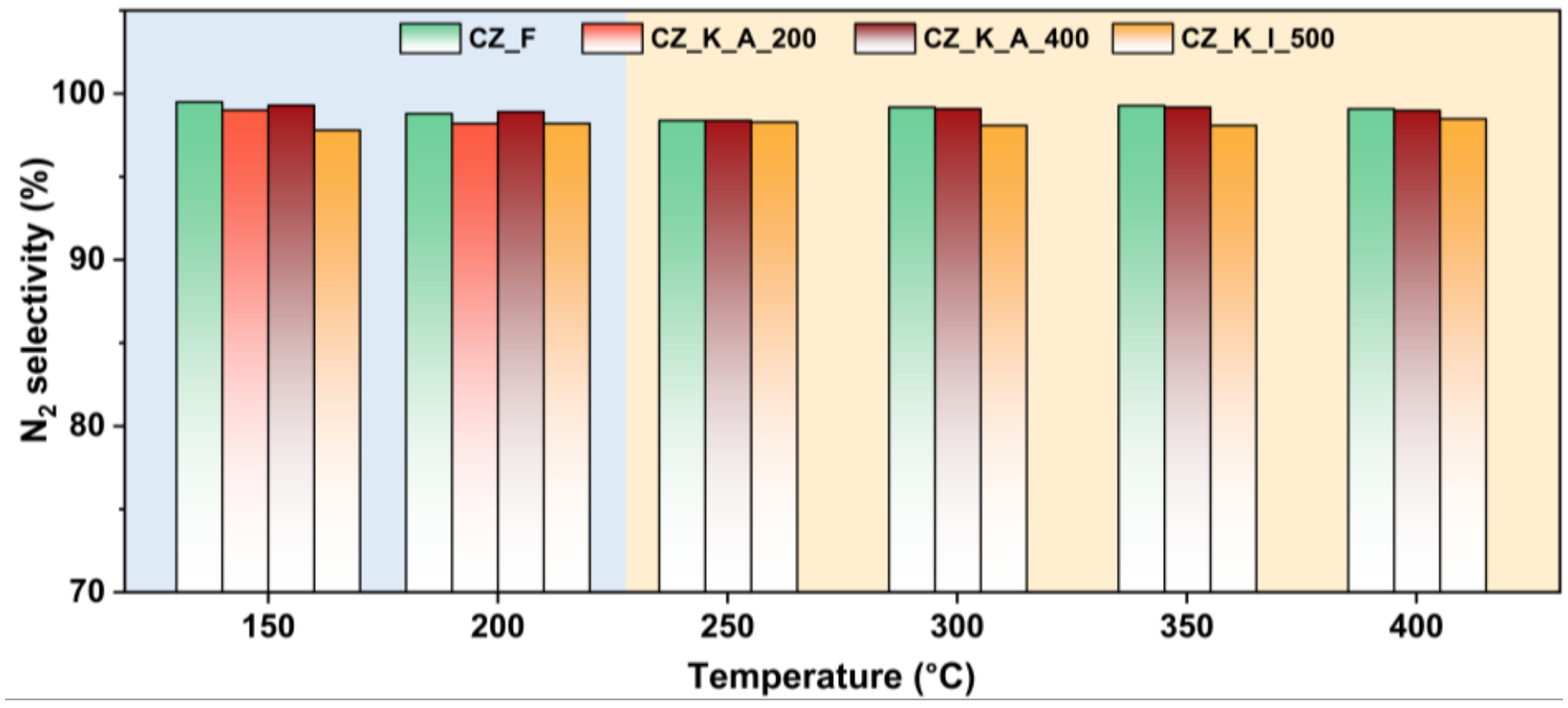

Figure S1. N_2 selectivity versus reaction temperature over fresh, aerosol-poisoned, and impregnated K-poisoned Cu/SSZ-13 catalysts.

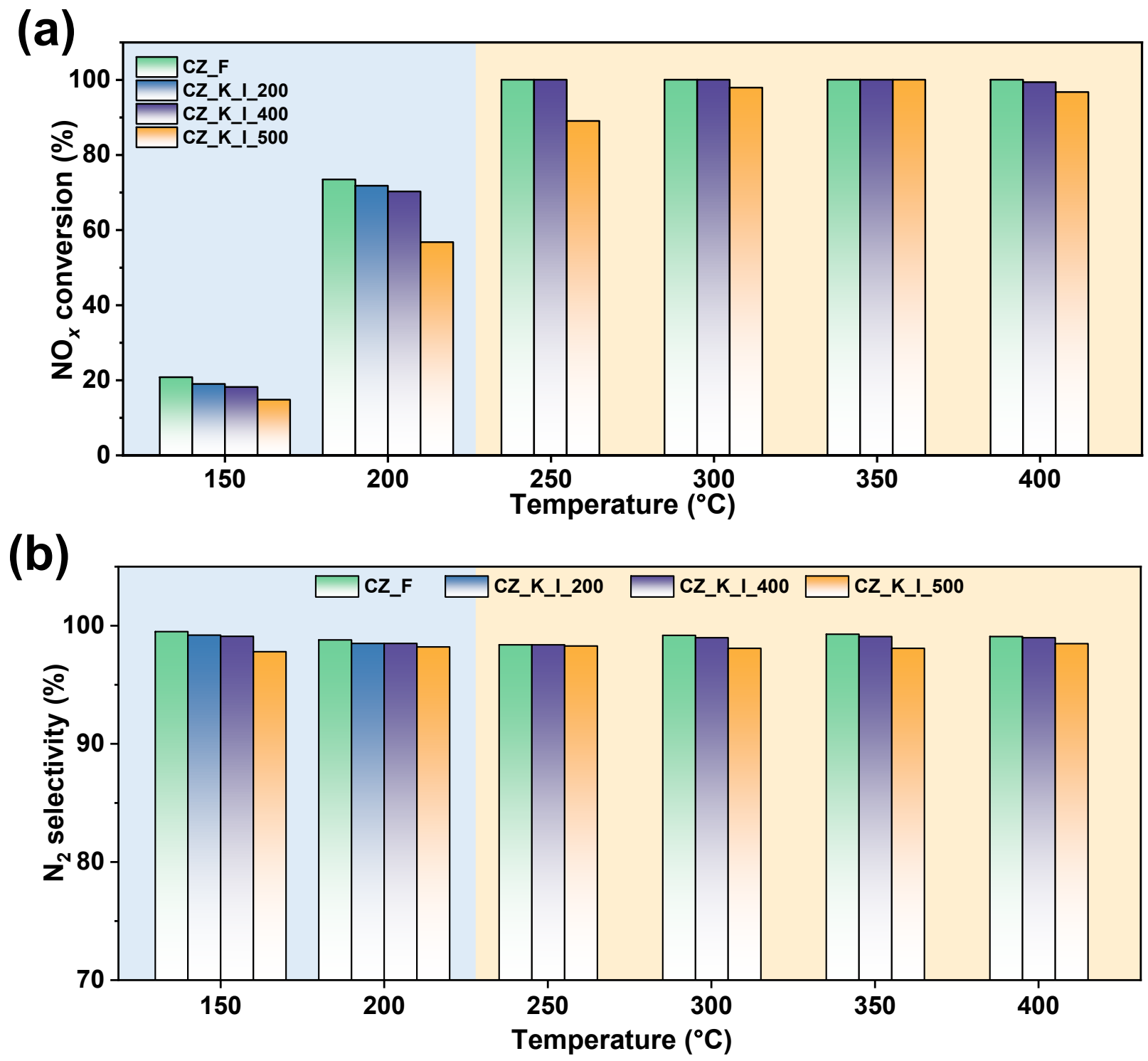

Figure S2. (a) NO_x conversion and (b) N_2 selectivity versus reaction temperature over fresh and impregnated K-poisoned Cu/SSZ-13 catalysts calcined at different temperatures.

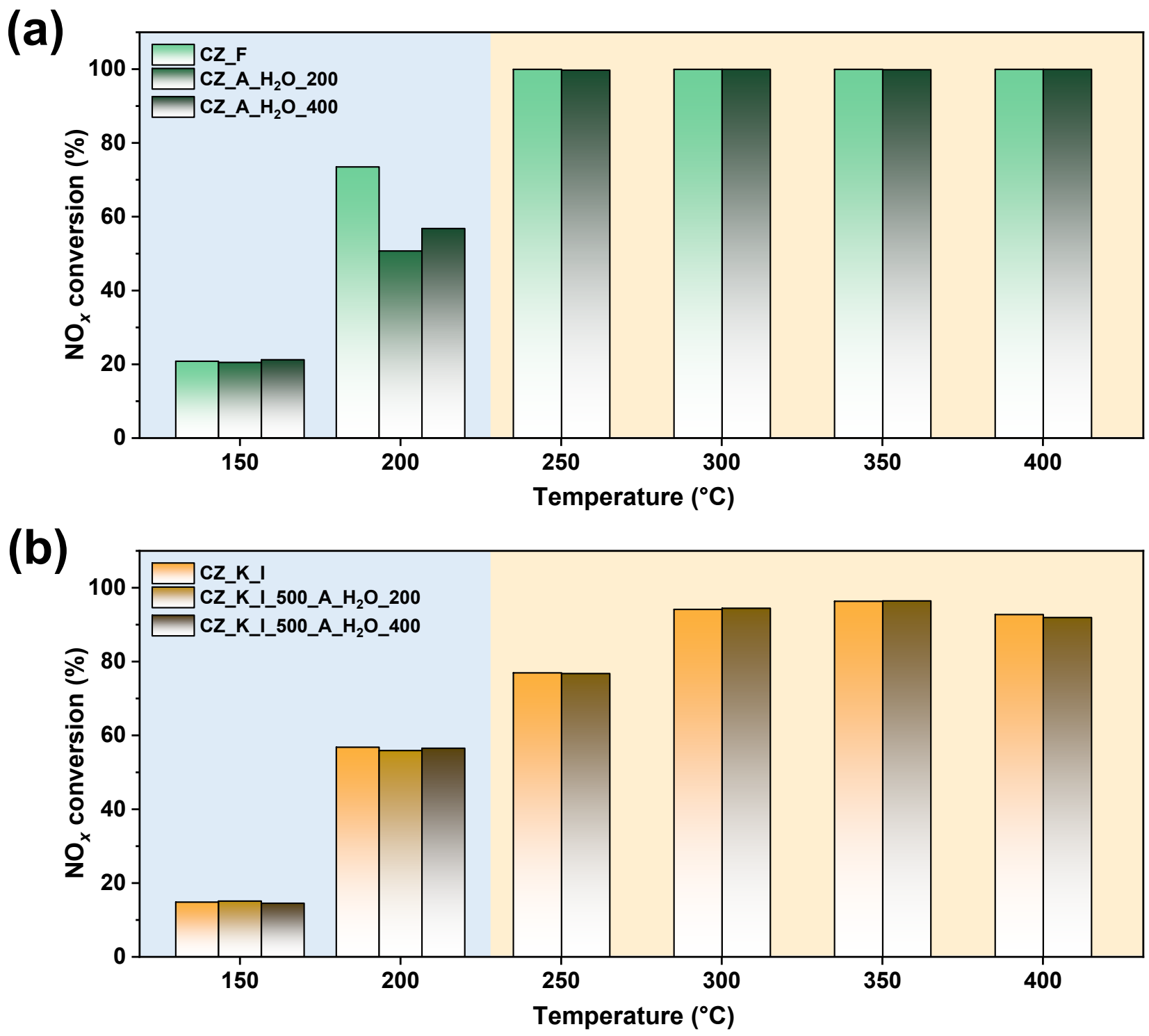

Figure S3. Effects of pure-water nebulization on the NO_x conversion of Cu/SSZ-13 catalysts. (a) Fresh Cu/SSZ-13 before and after 12 h treatment with nebulized pure water at 200 or 400 °C. (b) K-poisoned Cu/SSZ-13 prepared by impregnation before and after the same pure-water treatments.

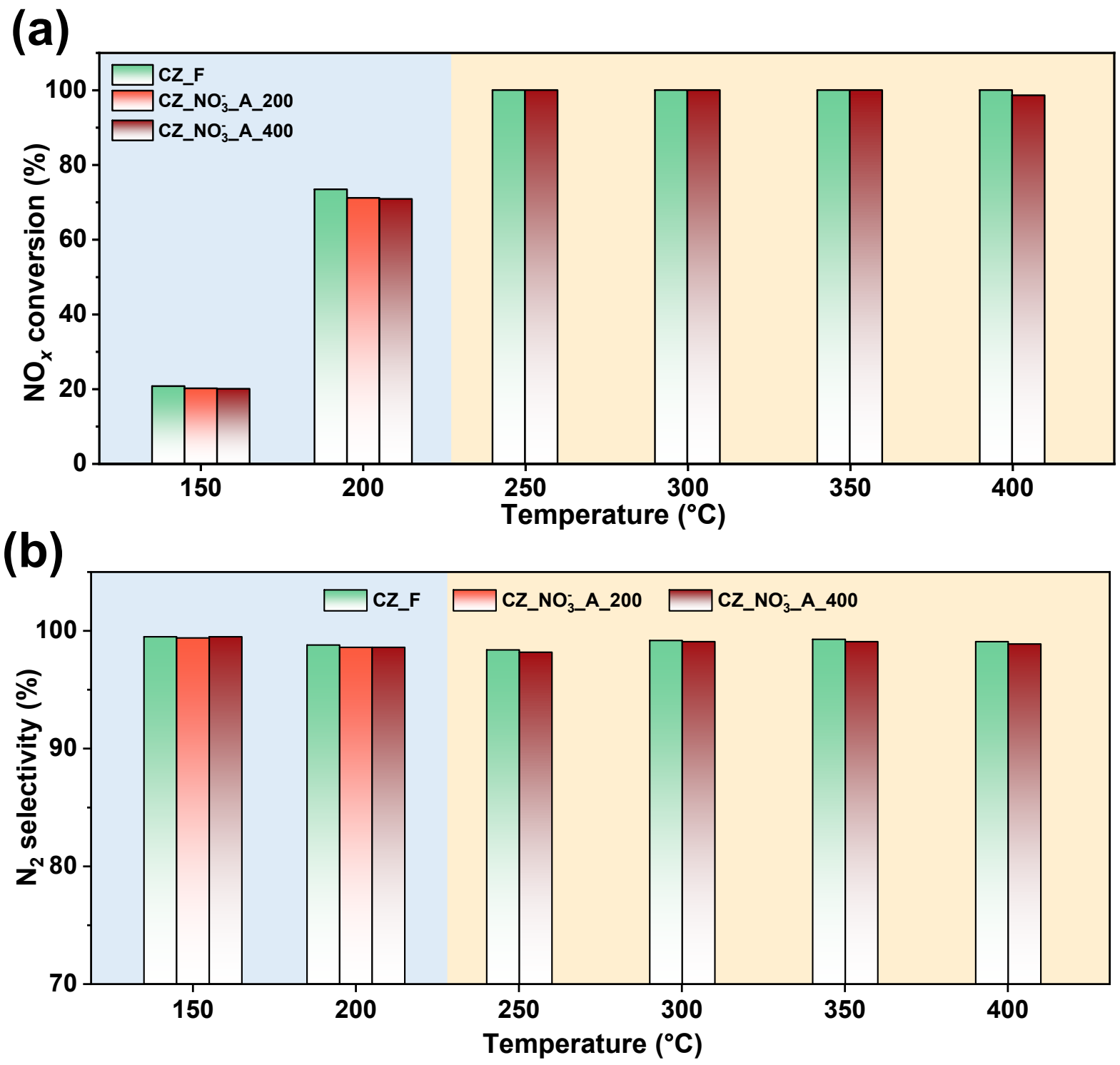

Figure S4. (a) NO_x conversion and (b) N_2 selectivity versus reaction temperature over fresh and NO_3^--treated Cu/SSZ-13 catalysts by aerosol-assisted treatment at 200 and 400 °C.

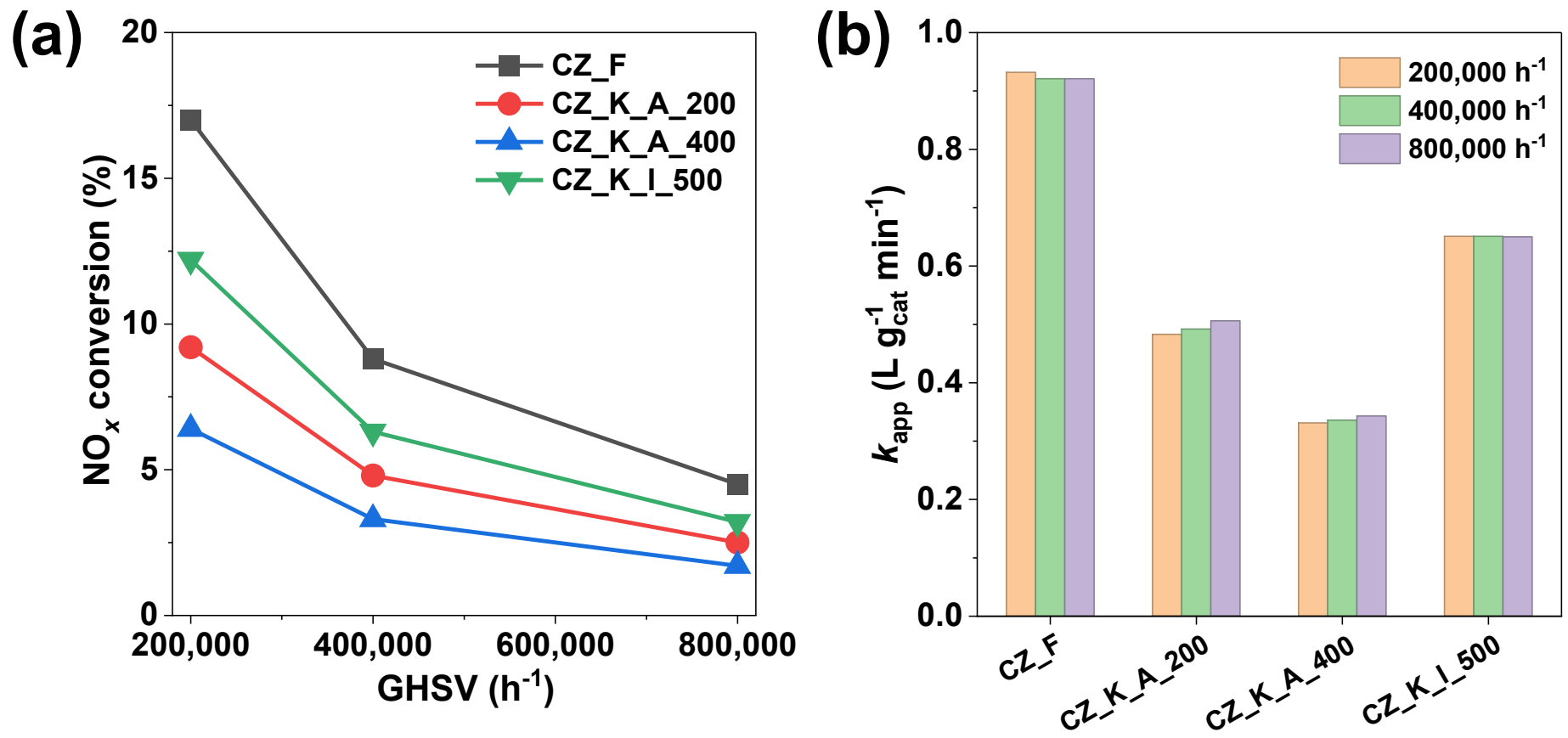

Figure S5. Effect of GHSV on the NH_3-SCR performance of fresh and K-poisoned Cu/SSZ-13 catalysts under low-conversion conditions. (a) NO_x conversion as a function of GHSV. (b) Apparent first-order rate coefficient, k_{app}, calculated from the measured NO_x conversions according to the plug-flow expression $k_{app} = -\left(\frac{F}{m}\right) \times ln(1-X)$,[1] where F is the total volumetric flow rate, m is the catalyst mass, and X is the NO_x conversion. Reaction conditions: 130 °C, 500 ppm NO, 500 ppm NH_3, 5% O_2, N_2 balance, and 0.02 g catalyst.

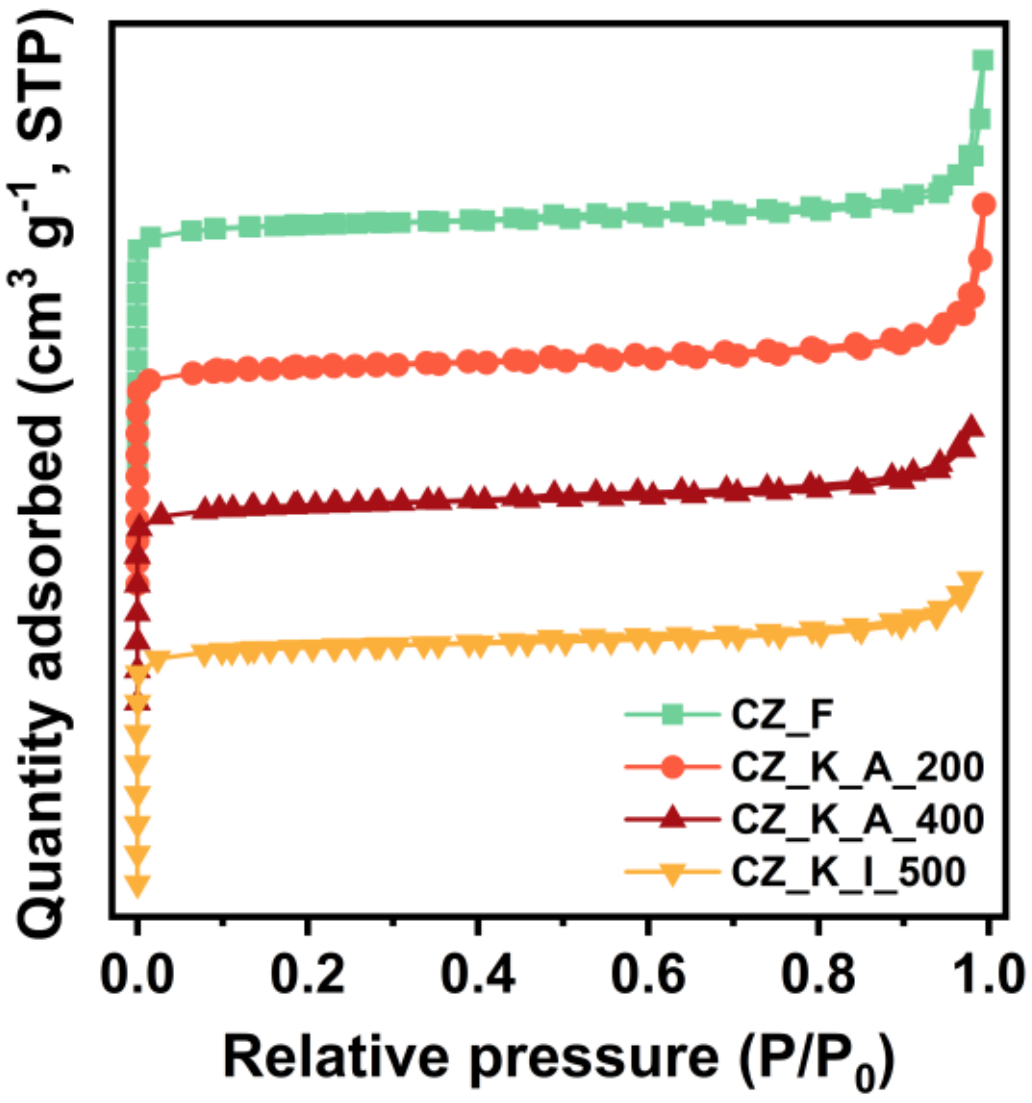

Figure S6. N_2 adsorption–desorption isotherms of fresh, aerosol-poisoned, and impregnated K-poisoned Cu/SSZ-13 catalysts.

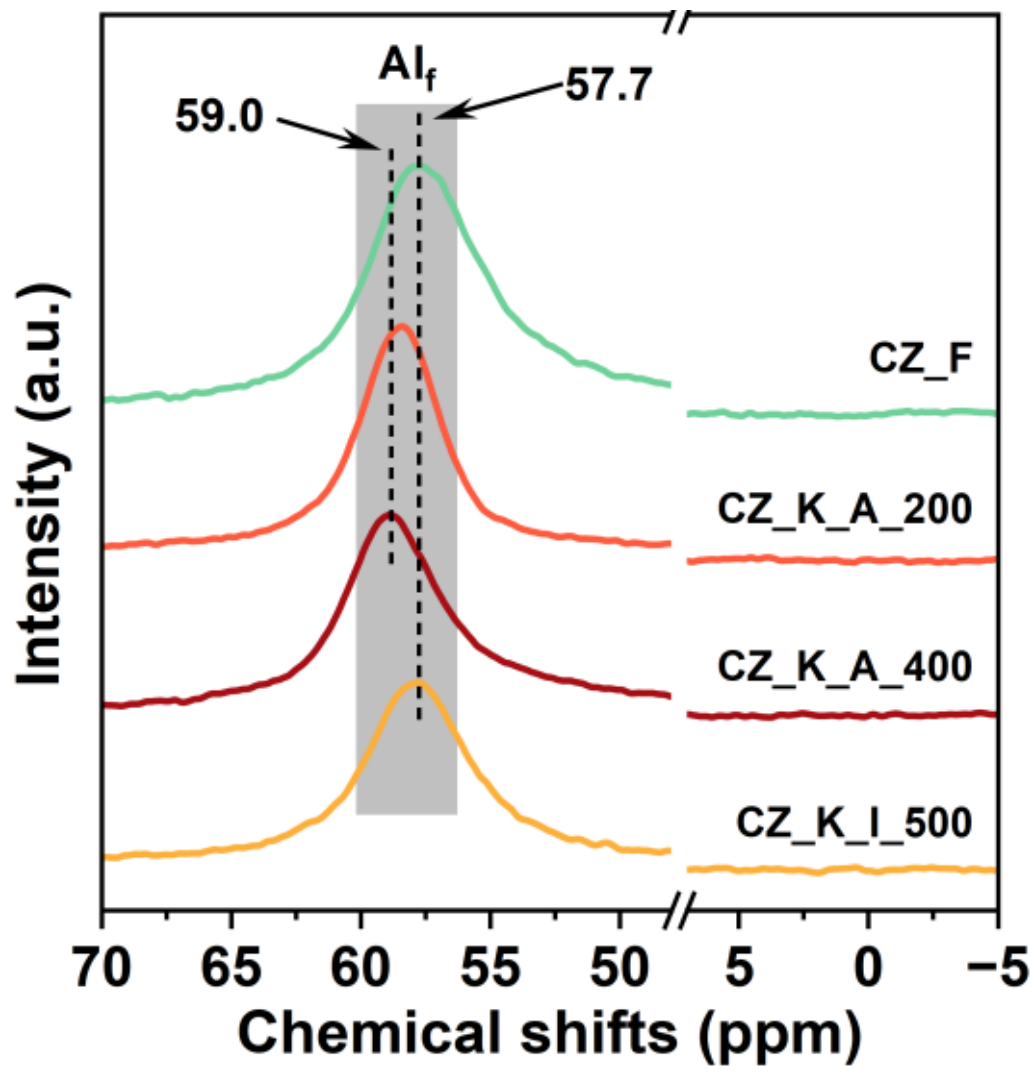

Figure S7. ^{27}Al MAS NMR spectra of the fresh, aerosol-poisoned, and impregnated K-poisoned samples.

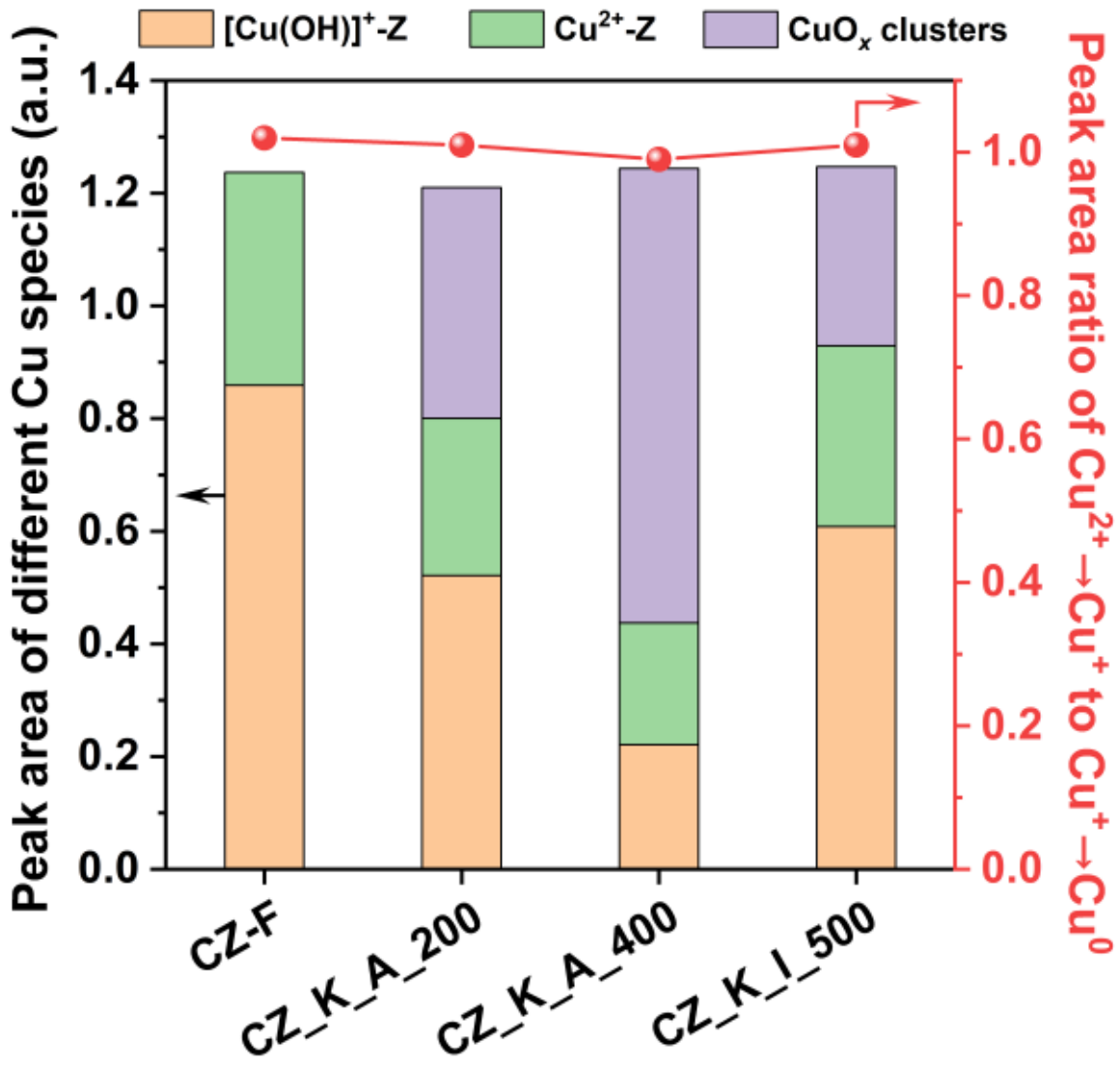

Figure S8. Quantitative Cu speciation from H_2-TPR peak deconvolution results (Table S1). The left y-axis shows peak areas of $[Cu(OH)]^{+}$–Z, Cu^{2+}–Z_2, and CuO_x clusters for fresh, aerosol-poisoned, and impregnated K-poisoned Cu/SSZ-13 catalysts; the right y-axis shows the corresponding reduction peak area ratios for the $Cu^{2+}\rightarrow Cu^{+}$ and $Cu^{+}\rightarrow Cu^{0}$ reduction steps.

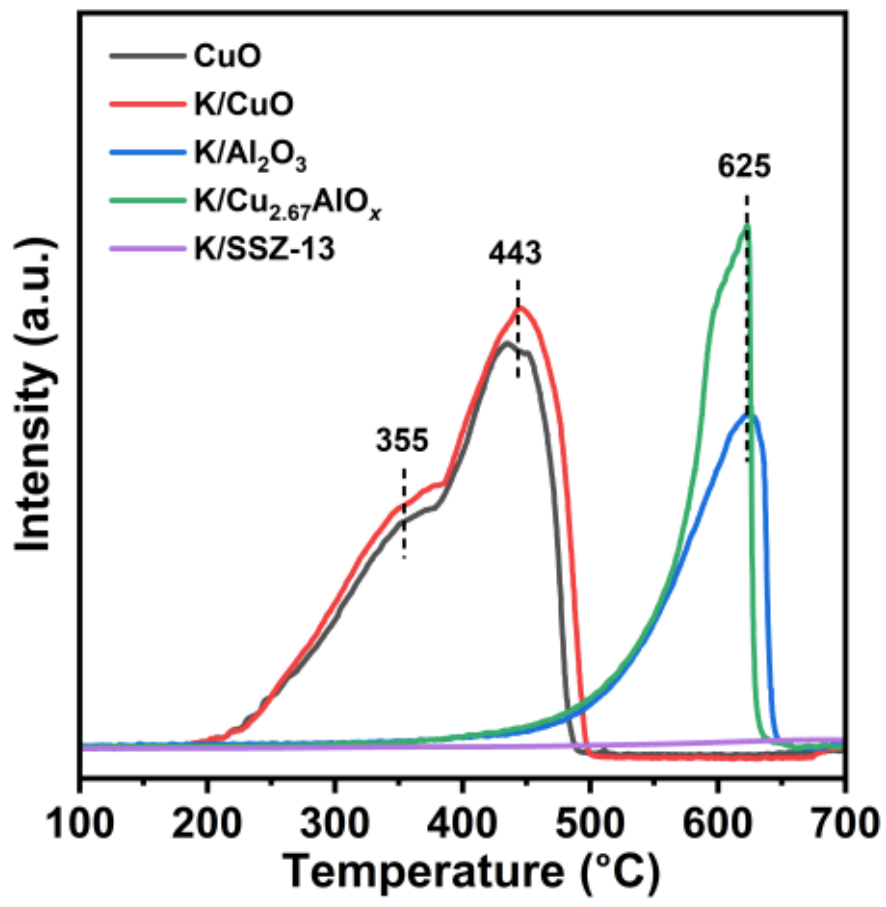

Figure S9. H_2-TPR profiles of CuO, K/CuO, K/Al_2O_3, K/$Cu_{2.67}AlO_x$, and K/SSZ-13 samples.

To assist the assignment of H_2-TPR reduction features of Cu/SSZ-13 before and after potassium poisoning in Figure 3b, reference CuO, Al_2O_3, and $Cu_{2.67}AlO_x$ samples were prepared by a co-precipitation method.[1] Zeolite H-SSZ-13 (Si/Al = 13.5) was provided by China Catalyst Holding Co., Ltd. Potassium-poisoned reference samples were also prepared by conventional incipient wetness impregnation.

As shown in Figure S9, potassium species themselves do not give rise to detectable H_2 reduction peaks. The H_2-TPR peaks observed at 355 and 443 °C for CuO and K/CuO, respectively, are assigned to the stepwise reduction of CuO to Cu^0. For K/Al_2O_3 and K/$Cu_{2.67}AlO_x$, the high-temperature reduction feature centered at 625 °C is consistent with the reduction of strongly interacting Cu-Al oxide domains (e.g., copper aluminate-like species) and/or highly stabilized CuO_x species on alumina, whereas Al_2O_3 itself is generally regarded as non-reducible under the present H_2-TPR conditions.[2] No H_2 reduction features are observed for H-SSZ-13, consistent with the absence of reducible metal oxide species.

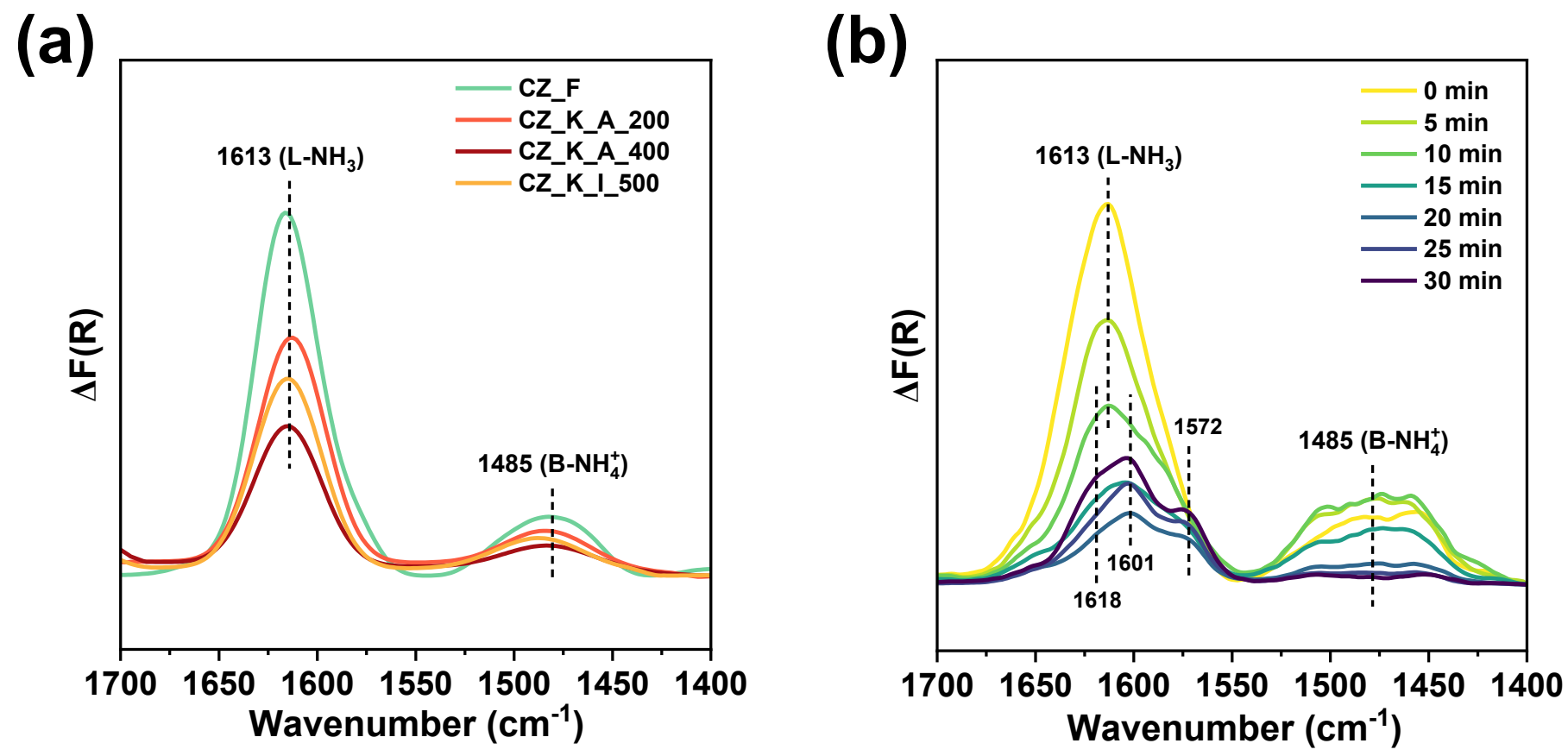

Figure S10. (a) NH_3-DRIFTS spectra of the fresh, aerosol-poisoned, and impregnated K-poisoned Cu/SSZ-13 catalysts. (b) DRIFTS spectra recorded after NH_3 saturation followed by NO + O_2 exposure for different times over fresh Cu/SSZ-13.

In Figure S10a, the bands at 1613 and 1485 cm^{-1} are assigned to deformation modes of NH_3 coordinated to Lewis acid sites (L-NH_3) and NH_4^+ species formed on Brønsted acid sites (B-NH_4^+), respectively.[3] After NH_3 saturation, switching the feed to NO + O_2 leads to a progressive decrease of both L-NH_3 and B-NH_4^+ species with increasing exposure time (Figure S10b). Concurrently, new bands emerge that are assigned to vibrational modes of adsorbed NO_2 (1618 cm^{-1}) and of bridging (1601 cm^{-1}) and bidentate (1572 cm^{-1}) nitrate species.[4]

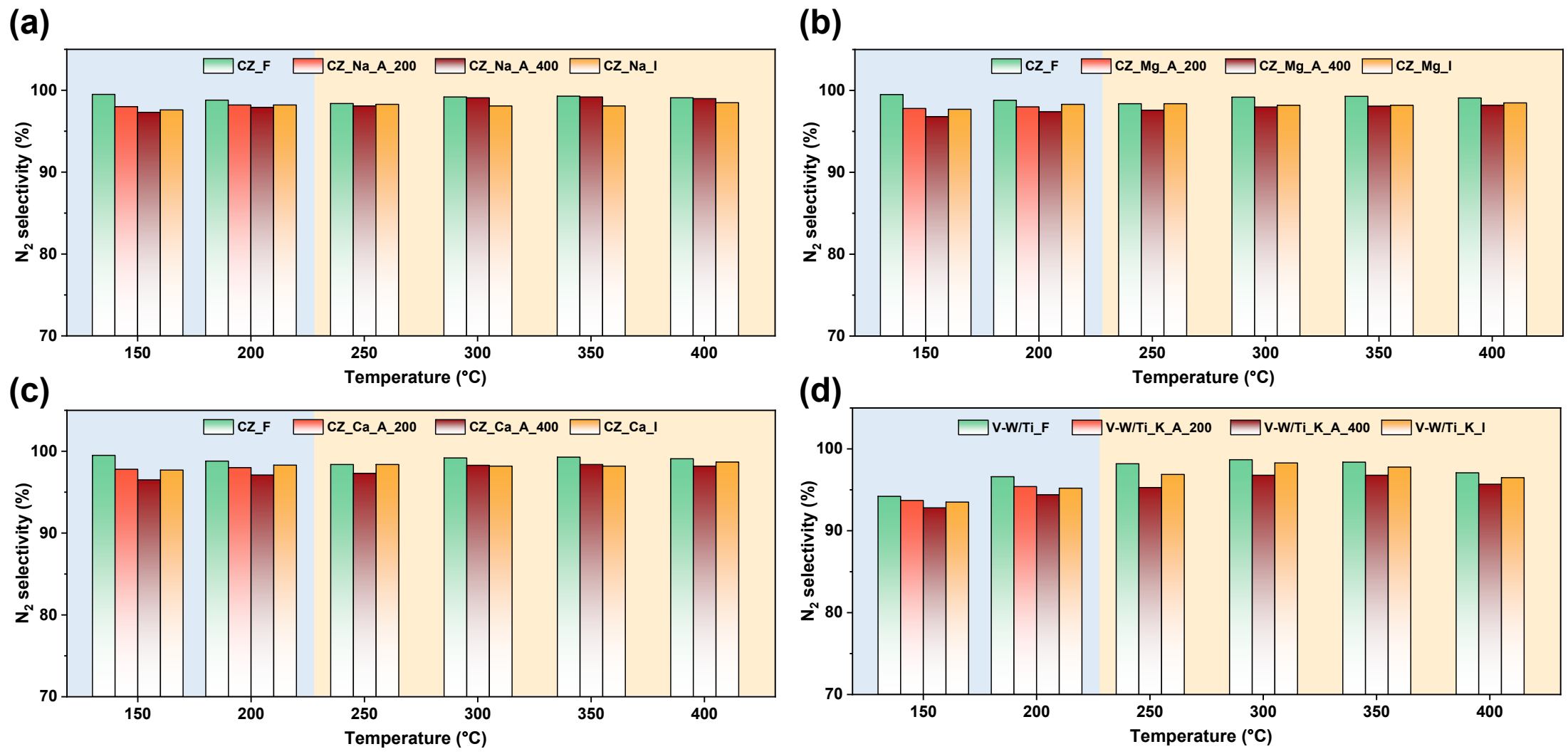

Figure S11. N_2 selectivity versus reaction temperature over fresh, aerosol-poisoned, and impregnated poisoned catalysts: (a) Na-, (b) Mg-, and (c) Ca-poisoned Cu/SSZ-13; (d) K-poisoned V_2O_5-WO_3/TiO_2.

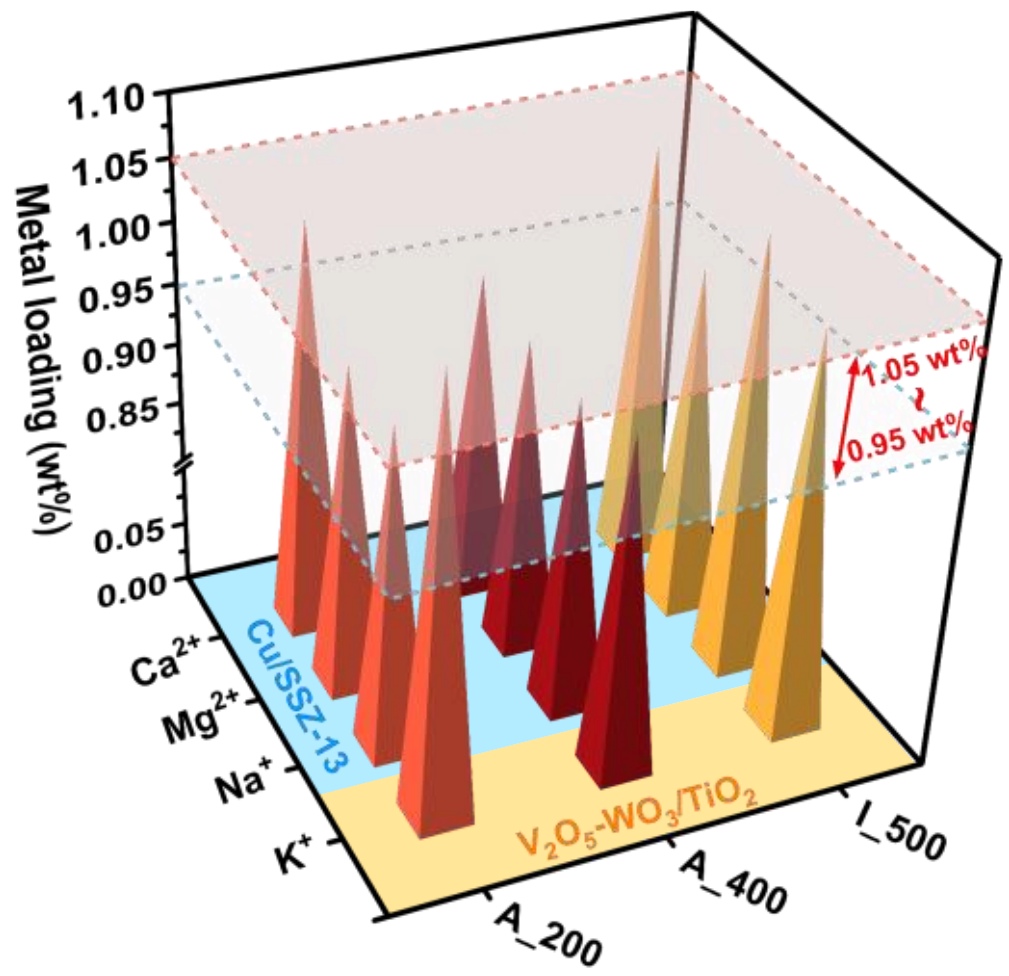

Figure S12. ICP-determined alkali and alkaline-earth metal contents in poisoned Cu/SSZ-13 and V_2O_5–WO_3/TiO_2 catalysts prepared by aerosol-assisted poisoning and impregnation methods.

As shown in Figure S12, for alkali- and alkaline-earth-metal-poisoned samples prepared with a target loading of 1 wt%, ICP analysis indicates that the actual metal contents fall within 0.95–1.05 wt%, confirming the good controllability and reliability of the aerosol-assisted, reaction-coupled poisoning protocol.

3. Supplementary Tables

Table S1. Textural properties, near-surface K concentration, and quantitative Cu speciation of the fresh, aerosol-poisoned, and impregnated K-poisoned samples.

Sample	S_{BET}[a] (m^2 g^{-1})	Near-surface K concentration[b] (at.%)	H_2-reduction peak areas[c]					
			$[(CuOH)]^+$-Z→Cu^+	Cu^{2+}-Z_2→Cu^+	CuO_x→Cu^0			Cu^+→Cu^0
CZ_F	602.4	-	0.859	0.378	0	0	0	1.209
CZ_K_A_200	529.1	0.46	0.521	0.279	0.137	0.378	0.305	0.789
CZ_K_A_400	461.4	0.38	0.221	0.216	0.319	0.675	0.62	0.439
CZ_K_I_500	547.0	0.60	0.608	0.321	0.170	0.397	0.069	0.923

[a] Determined by the BET method.

[b] Determined by XPS.

[c] Calculated from the H_2-TPR peak deconvolution shown in Figure 3b.

References

(1) Zhang, T.; Qu, R.; Su, W.; Li, J. A novel Ce–Ta mixed oxide catalyst for the selective catalytic reduction of NO_x with NH_3. *Appl. Catal. B: Environ Energy* **2015**, *176*, 338–346.

(2) San Pio, M.; Gallucci, F.; Roghair, I.; van Sint Annaland, M. On the mechanism controlling the redox kinetics of Cu-based oxygen carriers. *Chem. Eng. Res. Des.* **2017**, *124*, 193–201.

(3) Zhang, Y.; Zhu, H.; Zhang, T.; Li, J.; Chen, J.; Peng, Y.; Li, J. Revealing the synergistic deactivation mechanism of hydrothermal aging and SO_2 poisoning on Cu/SSZ-13 under SCR condition. *Environ. Sci. Technol.* **2022**, *56*, 1917–1926.

(4) Hadjiivanov, K. I. Identification of neutral and charged N_xO_y surface species by IR spectroscopy. *Catal. Rev.:Sci. Eng.* **2000**, *42*, 71–144.
